# Dopamine-enabled anti-Hebbian timing-dependent plasticity in prefrontal circuitry

**DOI:** 10.3389/fncir.2014.00038

**Published:** 2014-04-23

**Authors:** Hongyu Ruan, Taixiang Saur, Wei-Dong Yao

**Affiliations:** ^1^Harvard Medical School – New England Primate Research CenterSouthborough, MA, USA; ^2^Department of Psychiatry, Beth Israel Deaconess Medical CenterBoston, MA, USA

**Keywords:** STDP, Hebbian, dopamine, glutamate, reward, learning

## Abstract

Spike timing-dependent plasticity (STDP) of glutamatergic synapses is a Hebbian associative plasticity that may underlie certain forms of learning. A cardinal feature of STDP is its dependence on the temporal order of presynaptic and postsynaptic spikes during induction: pre–post (positive) pairings induce t-LTP (timing-dependent long-term potentiation) whereas post–pre (negative) pairings induce t-LTD (timing-dependent long-term depression). Dopamine (DA), a reward signal for behavioral learning, is believed to exert powerful modulations on synapse strength and plasticity, but its influence on STDP has remained incompletely understood. We previously showed that DA extends the temporal window of t-LTP in the prefrontal cortex (PFC) from +10 to +30 ms, gating Hebbian t-LTP. Here, we examined DA modulation of synaptic plasticity induced at negative timings in layer V pyramidal neurons on mouse medial PFC slices. Using a negative timing STDP protocol (60 post–pre pairings at 0.1 Hz, δt = -30 ms), we found that DA applied during post–pre pairings did not produce LTD, but instead enabled robust LTP. This anti-Hebbian t-LTP depended on GluN2B-containing NMDA receptors. Blocking D1- (D1Rs), but not D2- (D2Rs) class DA receptors or disrupting cAMP/PKA signaling in pyramidal neurons also abolished this atypical t-LTP, indicating that it was mediated by postsynaptic D1R-cAMP/PKA signaling in excitatory synapses. Unlike DA-enabled Hebbian t-LTP that requires suppression of GABAergic inhibition and cooperative actions of both D1Rs and D2Rs in separate PFC excitatory and inhibitory circuits, DA-enabled anti-Hebbian t-LTP occurred under intact inhibitory transmission and only required D1R activation in excitatory circuit. Our results establish DA as a potent modulator of coincidence detection during associative synaptic plasticity and suggest a mechanism by which DA facilitates input-target association during reward learning and top-down information processing in PFC circuits.

## INTRODUCTION

Spike timing-dependent plasticity (STDP) is a Hebbian synaptic learning rule that may underlie neural circuit remodeling and behavioral adaptations (Bi and Poo, 2001; Dan and Poo, 2006; Caporale and Dan, 2008; Feldman, 2012; Ganguly and Poo, 2013). In its canonical form, STDP depends on the temporal order and narrow window of presynaptic and postsynaptic spikes: pairings of pre–post spikes induce long-term potentiation (t-LTP) whereas post–pre spike pairings induce long-term depression (t-LTD; Magee and Johnston, 1997; Markram et al., 1997; Bi and Poo, 1998). At many synapses, induction of Hebbian STDP depends on postsynaptic *N*-methyl-D-aspartate receptors (NMDARs), a classical coincidence detector of presynaptic and postsynaptic discharges and a source of intracellular Ca^2^^+^ influx needed for synaptic modifications (Caporale and Dan, 2008; Feldman, 2012). Different NMDAR subunits may differentially contribute to STDP; for example, GluN2A and GluN2B subunits haven been shown to mediate t-LTP and t-LTD, respectively, in cultured hippocampal synapses (Gerkin et al., 2007), consistent with the different channel biophysics, synaptic localizations, and signaling mechanisms associated with these subunits (Riccio and Ginty, 2002; Cull-Candy and Leszkiewicz, 2004; Lau and Zukin, 2007). Opposite to classical Hebbian STDP, atypical forms of STDP have also been observed at some synapses, where pre–post spikings drive t-LTD and post–pre spikings drive t-LTP (Han et al., 2000; Fino et al., 2005; Safo and Regehr, 2005; Letzkus et al., 2006; Lu et al., 2007; Fino et al., 2008). These STDP variants, referred as anti-Hebbian, are relatively rare but also often depend on NMDARs, particularly anti-Hebbian t-LTP (Letzkus et al., 2006).

The quantitative rules of STDP are profoundly influenced by neuromodulations (Lin et al., 2003; Couey et al., 2007; Seol et al., 2007; Pawlak et al., 2010; Cassenaer and Laurent, 2012). A particularly important neuromodulator is dopamine (DA), believed to encode reward signal during behavioral reinforcement and learning (Schultz, 2002; Wise, 2004). Recent studies suggest that DA, via the activation of D1 (D1Rs)- and D2 (D2Rs)-class receptors, is required for STDP induction in striatal medium spiny neurons (Pawlak and Kerr, 2008; Shen et al., 2008). DA has also been shown to broaden the temporal window of t-LTP at hippocampal (Zhang et al., 2009) and prefrontal cortex (PFC; Xu and Yao, 2010) synapses and, remarkably, convert t-LTD into t-LTP in cultured hippocampal neurons. In both synapses, DA-driven extension of t-LTP timing window is mediated by postsynaptic D1R-cAMP/PKA signaling and is likely the result of a decreased t-LTP induction threshold (Zhang et al., 2009), suggesting an important role for DA in the control of associability of pre–post coincident stimuli that trigger STDP.

In many brain regions, LTP (including t-LTP) at glutamate synapses often cannot be induced when endogenous local GABAergic transmission is left unblocked, supporting a role for native GABAergic network in constraining the excitability and plasticity of excitatory circuits (Wigstrom and Gustafsson, 1983; Bissiere et al., 2003; Meredith et al., 2003; Liu et al., 2005). Interestingly, DA can remove the powerful inhibitory constraint in both lateral amygdala and medial PFC (mPFC), gating t-LTP induction at glutamate synapses on principle cells (Bissiere et al., 2003; Xu and Yao, 2010). The dopaminergic gating is mediated through a mechanism by which DA decreases GABA release by acting on D2Rs localized at presynaptic GABAergic terminals of a subset of PFC interneurons (Mrzljak et al., 1996; Chiu et al., 2010; Xu and Yao, 2010).

In this study, we investigated DA modulation of STDP in the mouse mPFC, an association cortex that mediates cognition, reward, and memory (Fuster, 2008). Much of these functions are regulated by DA and mediated by synaptic strength in PFC excitatory circuits (Seamans and Yang, 2004). We previously reported that DA, via cooperative activation of D2Rs in inhibitory circuits and D1Rs in excitatory circuits, enables t-LTP in layer V PFC pyramidal neurons over a positive timing window of 0 to +30 ms. We now extend our earlier work by examining DA modulation of STDP at negative timing. Our results indicate that DA drives t-LTP at -30 ms, enabling a form of anti-Hebbian t-LTP that depends on postsynaptic D1-cAMP/PKA signaling and GluN2B-containing NMDARs in pyramidal neurons. In contrast to the high susceptibility of Hebbian t-LTP to GABAergic inhibition, DA-enabled anti-Hebbian t-LTP can be induced under intact inhibitory transmission.

## MATERIALS AND METHODS

All procedures were conducted in accordance with the National Institutes of Health guidelines for the care and use of laboratory animals and with an approved IACUC protocol from the Harvard Medical Area Standing Committee on Animals. Coronal slices (300 μm) were cut from the mPFC (containing the anterior cingulate or prelimbic cortices) of C57BL/6J mice (postnatal day 30–50) with a Leica VT1200 vibratome (Xu et al., 2009; Xu and Yao, 2010). Slices were incubated at room temperature in oxygenated artificial cerebrospinal fluid (ACSF) containing (in mM) 126 NaCl, 2.5 KCl, 2.5 CaCl_2_, 1.2 MgCl_2_, 25 NaHCO_3_, 1.2 NaH_2_PO_4_, and 25 D-glucose for at least 1 h before electrophysiological recording. Slices were then transferred to a recording chamber and secured with a harp during recording.

Somatic whole-cell patch-clamp recordings were performed on individual layer V PFC pyramidal neurons using an Axoclamp 2B amplifier (Molecular Devices). All recordings were made at 32°C, maintained with a TC344 Dual Automatic Temperature Controller (Harvard Apparatus). Cells were visualized with an Olympus BX51WI upright microscope under infrared illumination and recognized by their pyramidal shapes. Presynaptic stimuli (0.033 Hz, 200 μs), where necessary, were delivered at superficial layers II/III with a concentric tungsten electrode (FHC). In current-clamp recordings, pipettes were filled with (in mM) 130 K-gluconate, 8 NaCl, 10 HEPES, 0.4 EGTA, 2 Mg-ATP, and 0.25 GTP-Tris, pH 7.25 (with KOH) and recordings were made at the resting membrane potential of the cell. Input resistance was monitored throughout the experiment from the voltage response to a -200 pA hyperpolarizing current. In voltage-clamp experiments, electrodes were filled with (in mM) 142 Cs-gluconate, 8 NaCl, 10 HEPES, 0.4 EGTA, 2.5 QX-314 [*N*-(2,6-dimethylphenylcarbamoylmethyl)triethylammonium bromide], 2 Mg-ATP, and 0.25 GTP-Tris, pH 7.25 (with CsOH). Neurons were voltage clamped at -60 or -30 mV unless specified otherwise. Picrotoxin, (2R)-amino-5-phosphonopentanoate (APV), MK-801, 6-cyano-7-nitroquinoxaline-2,3-dione (CNQX), NVP-AAM077, and ifenprodil, where indicated, were either included in ACSF throughout experiments or added after baseline recordings were established. DA at 100 μM (in the presence of 20 μM ascorbic acid) was made fresh on the day of experiments. Drugs (e.g., DA or its agonists/antagonists) applied during STDP induction were washed in approximately 4 min before the start of pre–post or post–pre spike pairings and washed out approximately 12 min thereafter with a gravity-driven perfusion system (Harvard Apparatus). For intracellular dialysis of PKI (6–22; PKA inhibitor 6–22 amide; Calbiochem), we waited for at least 10 min after the patch rupture to allow its diffusion to synapses. Signals were filtered at 1 kHz, digitized at 10–50 kHz, and analyzed with pClamp 9.2 (Molecular Devices) or Mini Analysis 6 (Synaptosoft).

All data are expressed as mean ± SEM. Statistical analysis was performed using unpaired Student’s *t*-tests or one-way ANOVA followed by Dunnett’s *post hoc* tests, as specified in individual figures.

## RESULTS

### DA ENABLES t-LTP IN NATIVE PFC CIRCUITS OVER A 60-ms TEMPORAL WINDOW

**FIGURE 1:**
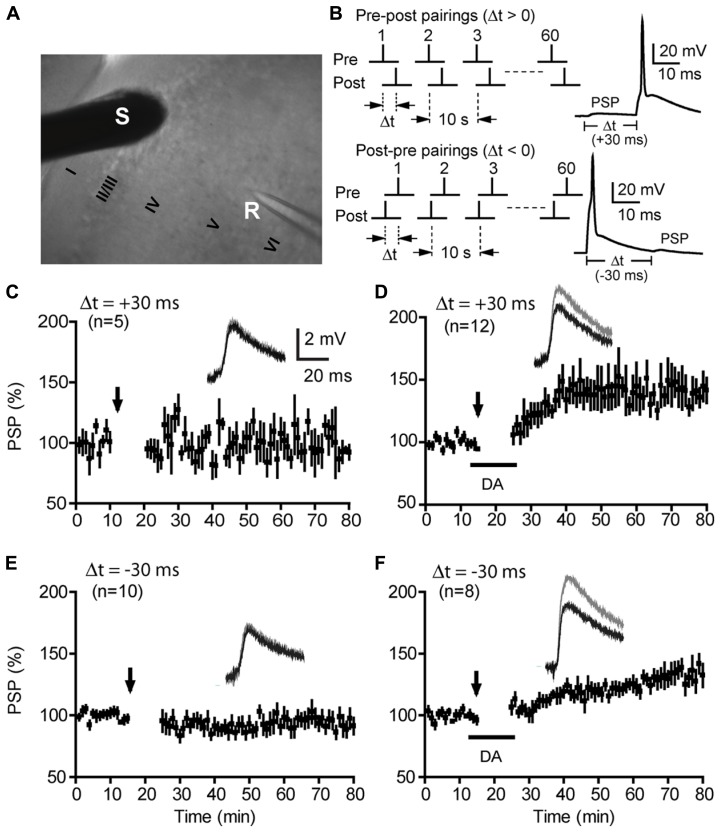
**Dopamine enables t-LTP over a 60-ms timing window under intact GABAergic conditions. (A)** A DIC image of a mouse mPFC coronal slice showing recording (R) and stimulation (S) sites. Cortical layers are also marked. **(B)** t-LTP induction protocols: paired presynaptic PSPs and postsynaptic APs with positive (top) and negative (bottom) Δt, delivered at 0.1 Hz for 10 min (60 pairs). Representative PSP-AP and AP-PSP responses during paired stimuli are shown. **(C,E)** Lack of t-LTP induction at Δt = +30 ms **(C)** or -30 ms **(E)** under control conditions (no DA). **(D,F)** Enabling of t-LTP by DA at Δt = +30 ms **(D)** or -30 ms **(F)**. Arrows indicate start of pre–post or post–pre pairings for t-LTP induction. DA (100 μM) was washed in approximately 4 min before the start of pairings and washed out approximately 12 min thereafter. Representative traces and scale bars are shown as insets. Values in parentheses indicate numbers of cells examined except as noted otherwise.

We performed whole-cell recordings from visually identified layer V pyramidal cells on mPFC slices (**Figure 1A**). Postsynaptic potentials (PSPs), evoked by extracellular stimuli at layer II/III, were recorded at the resting membrane potential (-67.8 ± 1.0 mV). This was nearly identical to the reversal potential of inhibitory postsynaptic currents (IPSCs) in this preparation (~-67 mV; Xu and Yao, 2010). At this resting level, PSPs were excitatory, mediated primarily by α -amino-3-hydroxy-5-methyl-4-isoxazolepropionic acid receptors (AMPARs), and with little contamination by inhibitory postsynaptic potentials (IPSPs) evoked as a result of excitation of local or feedforward inhibitory pathways (Xu and Yao, 2010).

Following a 10–15 min baseline recording, t-LTP was induced by 60 pairs (0.1 Hz) of presynaptically elicited PSPs and postsynaptic action potentials (APs) with variable pre–post (positive) or post–pre (negative) spike timing intervals (Δts; **Figure 1B**). The specificity, efficiency, and underlying mechanism of this STDP protocol to induce t-LTP at positive Δts have been established (Xu and Yao, 2010). Confirming our previous results, under conditions of unblocked GABAergic transmission (the GABA_A_ receptor blocker picrotoxin was omitted from the extracellular bath), pre–post pairings at Δt = +30 ms did not induce significant change in the amplitude of PSPs [105.7 ± 10.4%; *P* > 0.05 vs. baseline (101.5 ± 2.1%); **Figure 1C**]. However, when DA (100 μM) was added to the bath during pre–post pairings, the same protocol produced a lasting and significant increase in PSP amplitude (139.8 ± 6.4%; *P* < 0.01 vs. baseline; **Figure 1D**). Extending this finding to the negative Δt direction, we found that a classical t-LTD protocol (60 post–pre pairings, 0.1 Hz, Δt = -30 ms) did not induce LTD [93.5 ± 5.9%; *P* > 0.05 vs. baseline (99.8 ± 0.3%); **Figure 1E**], but instead induced a significant LTP [132.0 ± 1.3%; *P* < 0.05 vs. baseline (99.5 ± 0.9%); **Figure 1F**] when DA was applied to the extracellular bath during post–pre pairings. At a more extended negative timing interval (Δt = -50 ms), the presence of DA had no significant effect on the outcome of synaptic plasticity (Saur and Yao, data not shown). The DA-enabled t-LTP induced by post–pre pairings at -30 ms was not caused by a delayed potentiation of PSPs by DA itself because bath-applied DA in the absence of PSP-AP pairings produced a reversible depression of PSPs (Xu and Yao, 2010). In addition, DA had little effect on the intrinsic excitability of these neurons (Xu and Yao, 2010). This atypical form of t-LTP is opposite to the canonical Hebbian t-LTP driven by pre–post spike pairs, thus can be considered anti-Hebbian. Together, our data indicates that DA opens up a 60-ms temporal window (from -30 to +30 ms) that is otherwise closed for Hebbian and anti-Hebbian synaptic plasticity in native PFC circuits.

### DA-ENABLED ANTI-HEBBIAN LTP IS MEDIATED BY D1Rs, BUT NOT D2Rs, AND CAN BE INDUCED UNDER INTACT GABAergic TRANSMISSION BY D1R ACTIVATION ALONE

**FIGURE 2:**
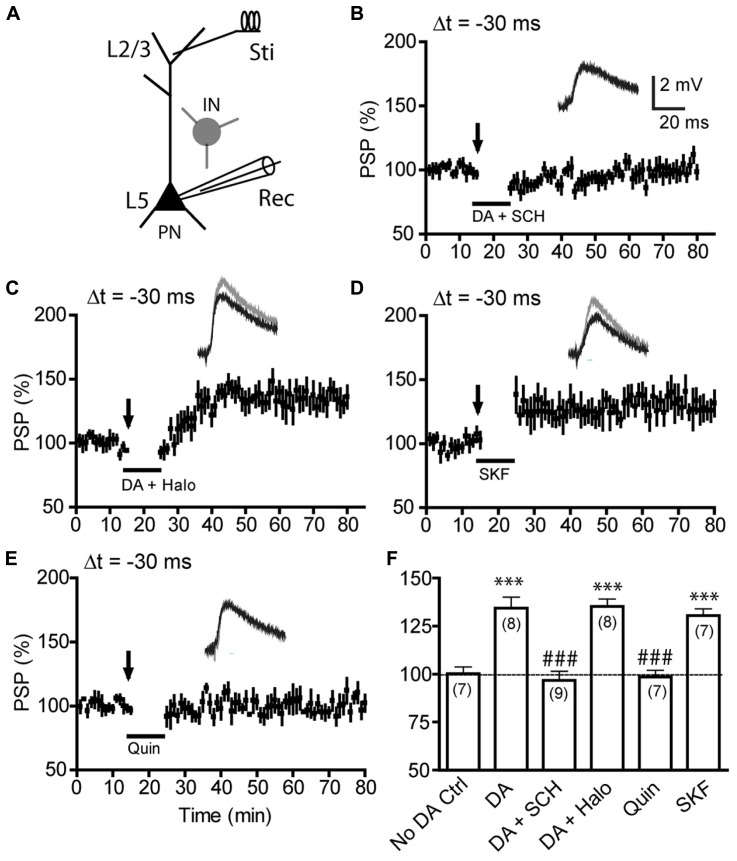
**Dopamine-enabled t-LTP at negative-timing depends on D1Rs, but not D2Rs and can be induced under intact GABAergic transmission by D1R, but not D2R, activation. (A)** All experiments in this figure were done with picrotoxin omitted in the bath to preserve GABAergic inhibitory transmission. **(B,C)** Effects of the D1R antagonist SCH23390 (SCH, 10 μM; **B**) and the D2R antagonist haloperidol (Halo, 2 μM; **C**) on DA-enabled t-LTP at Δt = -30 ms. DA enabled t-LTP was blocked by SCH23390 but not by haloperidol. **(D,E)** Effects of the D1R agonist SKF81297 (SKF, 2 μM; **D**) and the D2R agonist quinpirole (Quin, 10 μM; **E**) on t-LTP at Δt = -30 ms. SKF81297 alone, but not quinpirole alone, mimicked the effect of DA in enabling t-LTP under intact inhibition. **(F)** Summary of t-LTP induction under different conditions. ***P < 0.001 vs. No DA control; ^###^*P* < 0.001 vs. DA. Student’s *t*-tests.

We next investigated the DA receptor class(es) that mediate the negative-timing t-LTP (**Figure 2**). Under intact inhibitory transmission (**Figure 2A**), selective blockade of D1Rs by SCH23390 (10 μM; added to the perfusion bath 1 min before DA application) completely abolished the DA-enabled t-LTP at -30 ms (96.8 ± 4.6%; *P* > 0.05 vs. baseline; **Figures 2B,F**), suggesting a mandatory role for D1Rs in this t-LTP. In contrast, blocking D2Rs by including haloperidol (2 μM) during DA application failed to block this t-LTP (134.3 ± 6.1%; *P* > 0.05 vs. DA; **Figures 2C,F**), suggesting D2Rs did not contribute to this DA-enabled t-LTP. This result was unexpected because we and others had previously shown that DA-enabled t-LTP induced at positive timings requires activation of D2Rs when GABAergic transmission is left unblocked, through a mechanism by which DA acts on presynaptic D2Rs at local GABAergic terminals to suppress inhibitory transmission (Bissiere et al., 2003; Xu and Yao, 2010). Thus, our result suggests that DA-enabled t-LTP induction at -30 ms did not require suppression of the endogenous GABAergic inhibition. Indeed, application of the D1R agonist SKF81297 (2 μM) alone (129.2 ± 7.0%; **Figures 2D,F**) in the absence of picrotoxin was sufficient to mimic the effect of DA in enabling t-LTP at Δt -30 ms, whereas the D2R agonist quinpirole (10 μM) alone was insufficient (102.0 ± 4.4%; **Figures 2E,F**). Thus, like DA-enabled positive-timing t-LTP, DA-enabled negative-timing t-LTP is mediated by D1Rs; but unlike positive-timing LTP, the negative-timing t-LTP does not seem to be constrained by GABAergic transmission.

**FIGURE 3:**
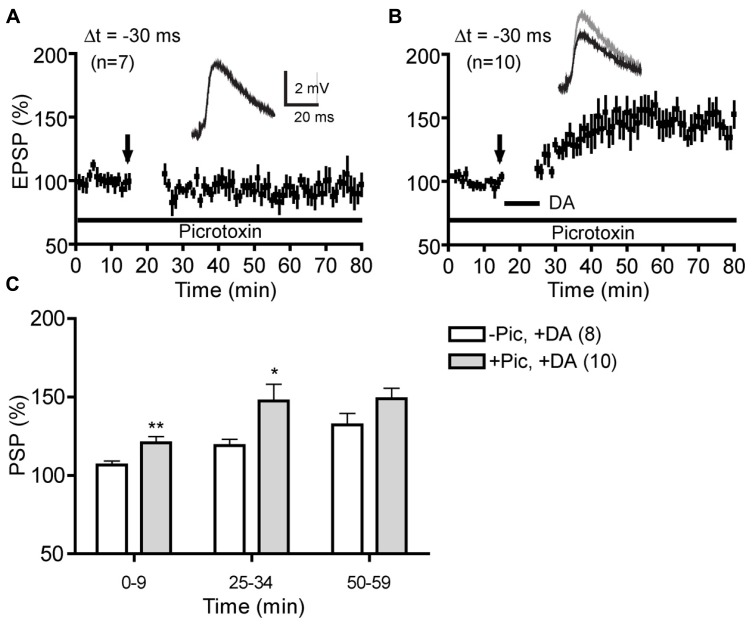
**Comparison of DA-enabled negative-timing t-LTP in the absence and presence of picrotoxin. (A)** Absence of t-LTP induction at Δt = -30 ms in control condition (no DA) in the presence of picrotoxin (50 μM).** (B)**DA-enabled t-LTP at Δt = -30 ms in the presence of picrotoxin. **(C)**Comparison of DA-enabled t-LTP magnitude with and without picrotoxin at different times following post–pre pairings. *P < 0.05; **P < 0.01. Student’s *t*-tests.

To further evaluate the role of GABAergic inhibition in negative-timing t-LTP, we compared the magnitude of DA-enabled -30 ms t-LTP in the absence and presence of picrotoxin at different time points following post–pre pairings (**Figure 3**). In the presence of picrotoxin, 60 pairs of postsynaptic AP and presynaptic EPSPs (excitatory PSPs) induced neither t-LTP nor t-LTD without bath-applied DA (95.2 ± 7.8%; **Figure 3A**), suggesting that this low-frequency, single-spike protocol was inefficient for LTD induction at -30 ms under control conditions. In contrast, when DA was supplied during pairings, this protocol induced robust t-LTP (146.0 ± 8.1%; **Figure 3B**). However, a direct comparison of this DA-enabled t-LTP with that in the absence of picrotoxin revealed a delayed occurrence of PSP potentiation when picrotoxin was omitted (**Figure 3C**). These experiments suggest some potential constraining effects of GABAergic inhibition on the development phase of t-LTP. Whether this was due to a transient potentiation of IPSPs following post–pre pairings that would shunt EPSPs or an inhibition of t-LTP induction/expression mechanism by GABAergic transmission remains to be determined. Nevertheless, our data suggest that Hebbian and anti-Hebbian t-LTP in the PFC depend on different DA receptor subtypes and display differential susceptibility to endogenous GABAergic circuit inhibition.

### DA-ENABLED ANTI-HEBBIAN LTP IS MEDIATED BY POSTSYNAPTIC D1R-cAMP/PKA SIGNALING IN PYRAMIDAL CELLS

**FIGURE 4:**
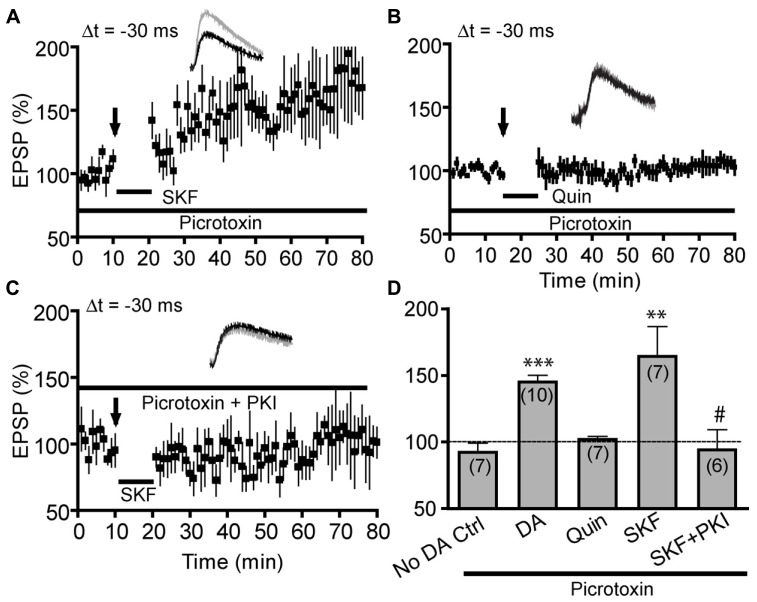
**Dopamine-enabled negative-timing t-LTP is mediated by postsynaptic D1-cAMP/PKA signaling in pyramidal cells.** Experiments in this figure were done in extracellular bath containing picrotoxin (50 μM). **(A)**SKF81297 (SKF, 2 μM) alone mimicked the effect of DA in enabling t-LTP at Δt = -30 ms. **(B)** Quinpirole (10 μM) alone failed to enable t-LTP at -30 ms. **(C)** SKF81297-enabled t-LTP was abolished by intracellular loading of PKI (6–22; 20 μM). **(D)** Summary of t-LTP at Δt = -30 ms under different conditions. **P < 0.01; ***P < 0.001 vs. No DA control; ^#^*P* < 0.05 vs. SKF. Student’s *t*-tests. Data for “No DA Ctrl” and “DA” was re-plotted from **Figures 3A,B** for direct comparison.

We next investigated the signaling mechanism underlying D1R-dependent anti-Hebbian t-LTP (**Figure 4**). Our previous study demonstrated that DA acts on D1Rs and downstream cAMP/PKA signaling in pyramidal neurons to drive t-LTP at Δt = +30 ms, an extended and normally ineffective timing interval (Xu and Yao, 2010). We hypothesized that similar signaling mechanism, i.e., postsynaptic D1R-cAMP/PKA pathway in excitatory synapses on pyramidal neurons mediates the anti-Hebbian t-LTP and thus studied SKF81297-enabled t-LTP at Δt = -30 ms in the presence of picrotoxin (50 μM): under these conditions, GABA_A_R-mediated inhibitory influence was blocked and effects of DA receptors were limited to excitatory synapses. Bath application of SKF81297 (2 μM) during post–pre pairings enabled significant t-LTP (162.9 ± 21.26%; **Figure 4A**), thus fully mimicking the enabling effect of DA (**Figure 4D**). As expected, quinpirole (10 μM) failed to enable t-LTP at -30 ms (100.9 ± 3.5%; **Figures 4B,D**), further supporting that D1Rs, but not D2Rs, in pyramidal cells of excitatory microcircuits mediate this negative-timing t-LTP. Importantly, loading postsynaptic neurons with PKI (6–22) (20 μM), a membrane-impermeable form of inhibitory peptide of PKA, completely abolished the SKF81297-enabled -30 ms t-LTP (94.18 ± 14.98%; **Figures 4C,D**), suggesting that this t-LTP depends on postsynaptic cAMP/PKA signaling. Taken together, our results indicate that, similar to DA-enabled Hebbian t-LTP at +30 ms, DA-enabled anti-Hebbian t-LTP at -30 ms depends on postsynaptic D1Rs and downstream cAMP/PKA signaling in pyramidal cells.

### DA-ENABLED ANTI-HEBBIAN t-LTP DEPENDS ON GluN2B-CONTAINING NMDARs

**FIGURE 5:**
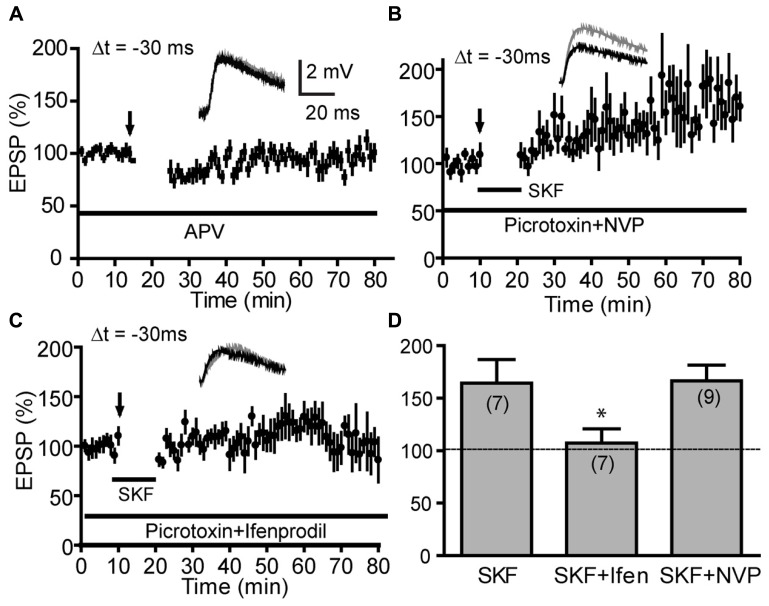
**Dopamine-enabled negative-timing t-LTP depends on GluN2B-NMDARs. (A)** DA-enabled t-LTP at Δt = -30 ms under intact GABAergic condition was blocked by bath-applied NMDAR antagonist APV (50 μM). **(B,C)**Effects of GluN2 subunit antagonists on SKF81297 (2 μM)-enabled t-LTP at -30 ms in the presence of picrotoxin (50 μM). The negative-timing t-LTP was blocked by bath-applied GluN2B antagonist ifenprodil (3 μM, **C**) but not by the GluN2A antagonist NVP-AAM077 (0.4 μM, **B**). **(D)** Summary of ifenprodil and NVP-AAM077 effects on SKF81297-enabled negative-timing t-LTP. SKF81297 data was re-plotted from **Figure 4F** for direct comparison. *P < 0.05, one-way ANOVA with Dunnett’s post-test vs. SKF control.

Conventional LTP and classical Hebbian t-LTP, including DA-enabled positive-timing t-LTP illustrated in our previous study (Xu and Yao, 2010), depend on postsynaptic NMDARs (Caporale and Dan, 2008). Including the NMDAR antagonist APV (50 μM) in the bath completely abolished DA-enabled t-LTP at -30 ms (96.9 ± 6.7%; **Figure 5A**), indicating that this anti-Hebbian t-LTP is also NMDAR-dependent. GluN2A and GluN2B subunits have been suggested to play differential roles in LTP and LTD (Liu et al., 2004; Massey et al., 2004) but see (Berberich et al., 2005; Weitlauf et al., 2005; Morishita et al., 2007). Thus, we further investigated which of these subunits might mediate DA-enabled negative-timing t-LTP, using ifenprodil, a GluN2B-specific inhibitor and NVP-AAM077, a GluN2A-preferred competitive antagonist (Auberson et al., 2002). Previous studies have shown that at 0.4 μM or lower, NVP-AAM077 selectively inhibits GluN2A-NMDAR-mediated currents in response to synaptically released glutamate in rodent hippocampal and PFC synapses (Weitlauf et al., 2005; Zhao et al., 2005; Gerkin et al., 2007). We found that at 0.4 μM, NVP-AAM077 did not prevent SKF81297-enabled t-LTP at -30 ms (166.4 ± 14.96%; **Figures 5B,D**), suggesting that GluN2A is not required to support this negative-timing t-LTP. In contrast, ifenprodil (3 μM; 107.2 ± 13.71%; **Figures 5C,D**) completely blocked SKF81297-enabled t-LTP at -30 ms, suggesting that the negative-timing t-LTP depended on GluN2B. Together, our analysis indicates that DA-enabled anti-Hebbian t-LTP is mediated by GluN2B-containing NMDARs.

### MODULATION OF SYNAPTIC GluN2A- AND GluN2B-NMDAR CURRENTS BY SKF81297

**FIGURE 6:**
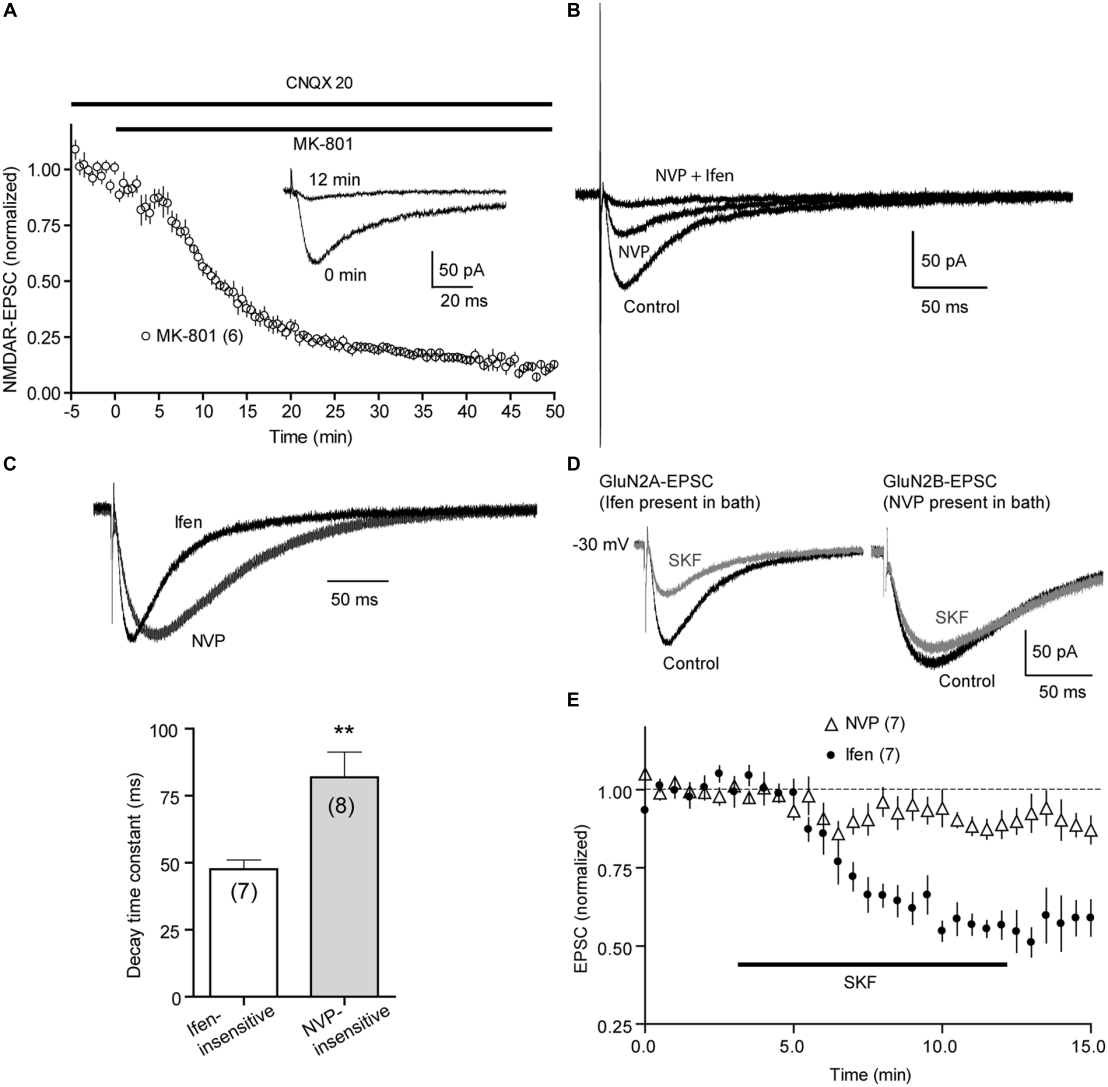
**Effects of SKF81297 on GluN2A- and GluN2B-mediated synaptic NMDAR currents. (A)** Progressive blockade of synaptic NMDAR current by MK-801. NMDA-EPSCs were recorded at -30 mV. Picrotoxin (50 μM) and CNQX (20 μM) were present in the bath to block GABA_A_ and AMPA receptor currents, respectively. MK-801 (20 μM) was added to the bath at 0 min. Insets, representative traces show NMDA-EPSCs at 0 and 12 min following MK-801 application. **(B)** PFC NMDA-EPSCs were composed primarily of GluN2A and GluN2B currents, as sequential addition of NVP-AAM077 (NVP; 0.4 μM) and ifenprodil (Ifen; 3 μM) abolished, most, if not all NMDA-EPSCs. Representative traces were shown. **(C)** Isolation of GluN2A- and GluN2B-mediated NMDAR currents. NVP-AAM077 or ifenprodil was included in the extracellular bath to isolate GluN2B- and GluN2A-mediated currents, respectively. Upper, representative traces were re-scaled and superimposed to compare their rise and decay kinetics. Lower, summary of average decay time constants of Ifen-insensitive and NVP-insensitive currents. Single-exponential fits were applied to the decay phase of currents to derive the decay time constant. **(D)** Representative traces show that bath-applied SKF81297 (2 μM) significantly suppress GluN2A (left; Ifen was present in bath), but not GluN2B currents (right; NVP was present in bath). Traces were taken approximated 3 min before and 10 min after SKF81297 application.** (E)** Summary time courses of SKF81297 effects on GluN2A- and GluN2B-mediated EPSCs. **P < 0.01, Student’s *t*-test.

GluN2A-NMDARs and GluN2B-NMDARs exhibit different channel conductance, kinetics, and subcellular localizations and are differentially required for t-LTP and t-LTD, respectively (Gerkin et al., 2007). A recent study also indicates that these NMDAR subtypes in the hippocampus are differentially modulated by D1Rs: GluN2B-NMDAR-mediated synaptic currents are potentiated whereas GluN2A-NMDAR currents are depressed (Varela et al., 2009). Because DA/D1R enables t-LTP at both +30 and -30 ms, normally ineffective timings, and GluN2B-NMDARs are required for DA/D1R-enabled t-LTP at -30 ms, it is possible that D1R activation enables t-LTP at these timings by enhancing GluN2B-NMDAR currents. To evaluate this possibility, we examined the modulation of synaptic GluN2A- and GluN2B-mediated NMDAR currents by D1R activation in PFC pyramidal neurons (**Figure 6**).

We recorded NMDAR-mediated excitatory postsynaptic currents (EPSCs) at -30 mV, a depolarized potential that permitted the removal of Mg^2^^+^ blockade of NMDAR channels. Picrotoxin (50 μM) and CNQX (20 μM) were included in the extracellular bath to block GABA_A_ receptor and AMPA receptor-mediated responses, respectively. EPSCs recoded under these conditions were mediated predominately by NMDARs as MK-801 (20 μM), an open channel NMDAR blocker, use-dependently inhibited synaptically evoked EPSCs (**Figure 6A**). In addition, the NMDAR-EPSCs were composed mainly of GluN2A and GluN2B currents, as sequential applications of NVP-AAM077 (0.4 μM) and ifenprodil (3 μM) nearly completely abolished the total NMDAR-EPSC (**Figure 6B**). Further supporting that GluN2A- and GluN2B-NMDAR currents were properly isolated, the NVP-AAM077-insensitive component (presumably GluN2B-NMDAR current) showed slower rise and decay compared to ifenprodil-insensitive component (presumably GluN2A-NMDAR current; **Figure 6C**).

Following a 5–10 min baseline recording, D1Rs were activated by adding SKF81297 (2 μM) to the bath for 10 min, a protocol similar to that for t-LTP induction. SKF81297 produced a sustained and significant suppression of GluN2A-EPSCs (64.16 ± 6.93 %; *P* < 0.05 vs. baseline), but a very modest, statistically insignificant reduction of GluN2B-EPSCs (89.94 ± 3.48%; *P* > 0.05; **Figures 6D,E**). This data suggests that D1R activation facilitates t-LTP at various timing intervals not by enhancing GluN2A or GluN2B-mediated NMDAR currents, and that additional signaling mechanism downstream of NMDAR-mediated Ca^2^^+^ influx must be involved.

### A CIRCUITRY-BASED MODEL OF DA MODULATION OF PFC SYNAPTIC PLASTICITY

**FIGURE 7:**
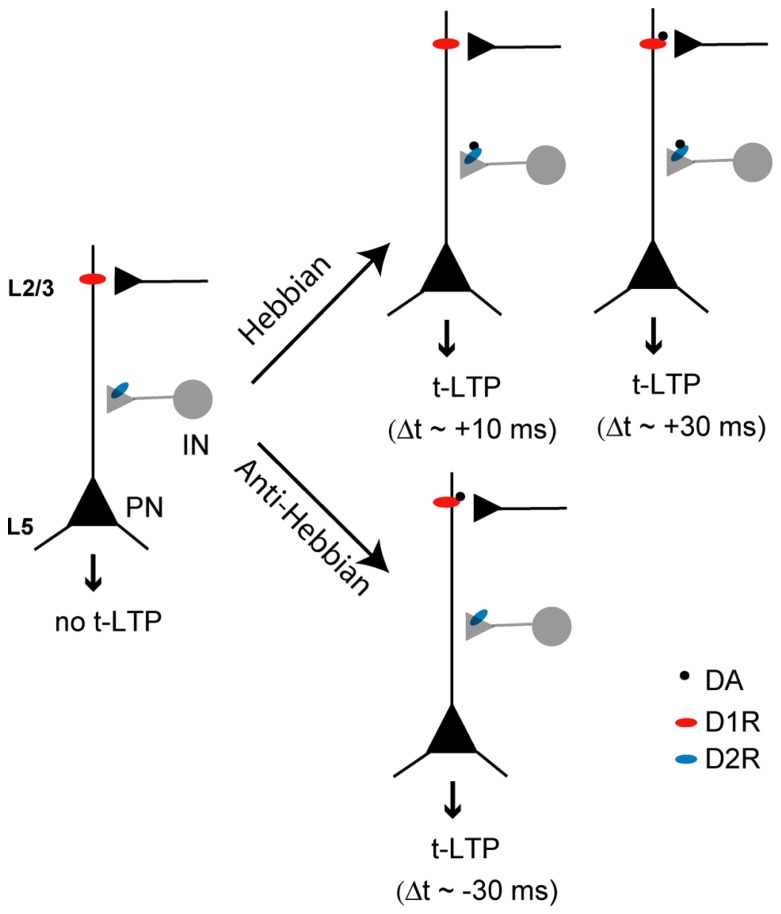
**A model for DA enabling of t-LTP in native PFC circuits.** t-LTP is absent when tissue DA level is minimal. When its concentration rises, DA can gate Hebbian t-LTP across a timing window of 0 → +30 ms. However, the mechanisms of t-LTP induction at different timings vary: at Δt = +10 ms, DA gates t-LTP induction through suppression of presynaptic GABA release by activating D2Rs at GABAergic terminals. At Δt = +30 ms, DA gates t-LTP induction through both suppression of presynaptic GABA release via D2Rs and postsynaptic activation of cAMP/PKA signaling downstream to D1Rs, highlighting the need of concurrent activation of both D1Rs and D2Rs in separate excitatory and inhibitory circuits. In contrast, negative-timing t-LTP can be gated by DA as well, but this form of anti-Hebbian t-LTP can be induced by activating postsynaptic D1Rs alone without the need to suppress GABAergic transmission involving presynaptic D2Rs in inhibitory circuits. Consequently, circuit cooperativity is not necessary for DA-enabled anti-Hebbian t-LTP. PN, pyramidal neurons; IN, interneurons; L2/3, layer 2/3; L5, layer 5.

In summary, combined with our previous work (Xu and Yao, 2010), the above experiments support a working model by which DA drives both Hebbian and anti-Hebbian t-LTP in native PFC circuits (**Figure 7**). Under resting physiological conditions where GABAergic transmission is intact and basal (tonic) DA level is low, no t-LTP can be elicited in layer V output neurons. When DA level rises (as is expected during attentional or motivational arousal), t-LTP is enabled across a temporal window that ranges from -30 to +30 ms. DA suppresses inhibitory transmission by acting at D2Rs on GABAergic terminals to gate positive-timing Hebbian t-LTP. This D2R-mediated disinhibition alone is sufficient to drive t-LTP at Δt = +10 ms. However, induction of t-LTP at +30 ms, a substantially extended, normally ineffective positive timing also requires activation of postsynaptic D1R-cAMP/PKA pathway in pyramidal neurons, suggesting a need for cooperative actions of D1Rs and D2Rs in separate inhibitory and excitatory microcircuits. In contrast, DA-enabled t-LTP at -30 ms requires only the activation of postsynaptic D1R-cAMP/PKA signaling in excitatory microcircuits, regardless of the presence of endogenous GABAergic inhibition. Thus, DA “opens” a 60 ms timing window that is otherwise “closed” for associative synaptic plasticity in prefrontal circuits.

## DISCUSSION

### POSTSYNAPTIC D1 RECEPTORS AS COINCIDENCE MODULATORS

The present study highlights a profound modulation of STDP quantitative rule by DA in the mouse PFC. The results support the notion that postsynaptic D1Rs, coupled to downstream cAMP/PKA signaling, are potent modulators of coincidence detection during associative synaptic plasticity. The normal temporal window for t-LTP induction in PFC excitatory synapses is approximately 10 ms (0 to +10 ms), which is extended by DA to +30 ms (Xu and Yao, 2010) and -30 ms (this study), resulting in a six-fold broadening! As in other synapses, NMDARs mediate DA-enabled t-LTP at both positive and negative timings across the window in these PFC synapses. However, activation of D1Rs by SKF81297 suppresses, rather than potentiates, both GluN2A- and GluN2B-mediated NMDAR currents. This result suggests that DA extends t-LTP window not by modulating NMDAR channels *per se*, but by acting on downstream signaling mechanisms that control t-LTP induction, similar to that seen in hippocampal neurons (Zhang et al., 2009).

The D1R-mediated inhibition of GluN2A-NMDARs and GluN2B-NMDARs contrasts the result from CA1 pyramidal cells in the mouse hippocampus, where these currents are oppositely regulated by D1Rs (Varela et al., 2009). Brain region differences in NMDAR compositions (Zhao et al., 2005; Wang et al., 2008) and DA signaling details, as well as variations in experimental conditions might contribute to the discrepancy. The suppression of both GluN2A-NMDAR and GluN2B-NMDAR currents by SKF81297 seems surprising because previous studies have shown that low-concentration SKF81297 potentiates synaptic NMDAR-EPSCs (Seamans et al., 2001). However, DA modulation of NMDARs in the PFC is known to be complex, and many factors, including drug types and concentrations, influence the result (Seamans and Yang, 2004). For example, SKF81297 is known to exert an inverted-U dose-dependent modulation of NMDAR activity, where low doses potentiate but high doses inhibit it (Seamans and Yang, 2004). It would be important in the future to further determine the factors that contribute to D1 modulation of PFC NMDARs under different conditions.

What downstream mechanisms might be targeted by DA to drive t-LTP at negative timings? The dependence of -30 ms t-LTP on GluN2B-NMDARs, but not GluN2A-NMDARs, indicates that DA acts on GluN2B-mediated cellular signaling. Perhaps due to their unique subcellular localization, i.e., extrasynaptic (Bliss and Schoepfer, 2004; which is yet to be confirmed in the PFC by ultrastructural studies), GluN2B-NMDARs have been considered especially suitable for detection of post–pre spiking pairs, transducing negatively correlated synaptic activity patterns to LTD (Gerkin et al., 2007). Compared to GluN2A-NMDARs, GluN2B-NMDARs undergo a slower Mg^2^^+^ unblockade by back-propagating APs (bAPs; Clarke and Johnson, 2006), have a lower open channel probability (Chen et al., 1999), and permit less Ca^2^^+^ influx, favoring the induction of LTD possibly by activating protein phosphatases 1 (PP1) and 2B (PP2B/calcineurin; Mulkey et al., 1994; Morishita et al., 2001). DA can inhibit PP1 and activate CaMKII, an essential signaling molecule required for most forms of LTP (Malenka and Bear, 2004), in the synapse through the D1R-cAMP/PKA-Inhibitor I/DARPP-32 pathway (Greengard et al., 1999), thus converting a “would-be-LTD” elicited by negative timing stimuli to LTP. Not necessarily mutually exclusive, D1R-cAMP/PKA signaling could also modulate voltage-sensitive dendritic ion conductances (Seamans and Yang, 2004) to influence the non-linear interaction of bAPs and subsequent EPSPs (Johnston et al., 1999), generating a Ca^2^^+^ influx patterns that favor t-LTD. Regardless of the mechanisms, our data indicate that DA has a potent role in postsynaptic co-incidence detection during STDP, markedly broadening the temporal window for timing-dependent LTP induction.

### HEBBIAN vs. ANTI-HEBBIAN t-LTP IN PFC CIRCUITS

In Hebb’s (1949) original postulate, a lasting increase in synaptic strength occurs if repeated presynaptic firing precedes and contributes to firing of postsynaptic cells. The canonical form of STDP, especially the “LTP arm” is considered Hebbian because plasticity is induced by repeated pairings of pre–post discharges. In this regard, the DA-enabled t-LTP at -30 ms in our study is “anti-Hebbian.” Similar forms of anti-Hebbian t-LTP have also been observed at several other synapses, including distal synapses between layer II/III and V pyramidal neurons in the somatosensory cortex (Letzkus et al., 2006), excitatory synapses onto striatal medium spiny neurons and cholinergic interneurons (Fino et al., 2005, 2008), and synapses between cultured hippocampal neurons (Zhang et al., 2009). Importantly, the anti-Hebbian t-LTP described here and elsewhere (Letzkus et al., 2006) depends on activation of postsynaptic NMDARs, suggesting that it is still associative by nature. This STDP variant sharply contrasts a non-associative, NMDAR-independent form of anti-Hebbian LTP in hippocampal interneurons that depends on hyperpolarization and Ca^2^^+^-permeable AMPARs (Lamsa et al., 2007).

Our protocol for anti-Hebbian t-LTP involves pairing 60 post–pre spikes at 0.1 Hz with Δt = -30 ms, a straightforward correlate of our t-LTP protocols (60 pre–post pairs at 0.1 Hz, +10 to +30 ms). Interestingly, while the positive-timing protocols are effective in inducing robust LTP in the absence of DA, the negative-timing protocol is ineffective in inducing LTD. Given that similar negative-timing protocols are effective in LTD induction in other DA target areas (Pawlak et al., 2010), the inability of our protocol to induce LTD in the PFC is surprising. Our data suggests that PFC plasticity mechanisms are rather unique. Future studies are needed to establish effective t-LTD protocols in the PFC under control conditions and it will be interesting to see whether such t-LTD can be converted to anti-Hebbian by DA, as is the case for hippocampal synapses (Zhang et al., 2009).

Our study provides evidence that Hebbian and anti-Hebbian t-LTP are differentially regulated by GABAergic inhibitory circuits. LTP, both conventional high-frequency stimulation (HFS)-induced and positive timing-dependent, is susceptible to GABAergic inhibition (Wigstrom and Gustafsson, 1983; Bissiere et al., 2003; Meredith et al., 2003; Liu et al., 2005; Tully et al., 2007), suggesting that Hebbian LTP is constrained by inhibitory network under native conditions. Consistent with this view, we recently showed that the induction of positive-timing t-LTP in PFC layer V neurons requires suppression of GABAergic transmission (Xu and Yao, 2010). In contrast, our current findings indicate that negative-timing t-LTP can be induced, albeit with a more delayed time course, without suppressing endogenous inhibitory transmission, suggesting that GABAergic circuits have a less constraining effect on anti-Hebbian t-LTP. The differential effects of GABA on Hebbian and anti-Hebbian t-LTP may be attributed to differences in the timing of GABA release in pre–post and post–pre pairings. In our experiments, GABA release is likely associated with activation of the cortical feedforward inhibitory pathway by presynaptic layer II/III stimulation. Although GABA is unlikely to influence dendritic membrane properties at resting state because of the near identical resting membrane potential and Cl^-^ reversal potential in PFC pyramidal neurons, it may differentially impact dendritic depolarization during pre–post or post–pre pairings. Specifically, GABA-mediated IPSPs shunt EPSPs on the rising phase of bAPs during pre–post pairings whereas IPSPs curtail EPSPs on the falling tail of bAPs during post–pre pairings. As a consequence, GABA exerts different effects on EPSP, bAP, and their non-linear summation under the two timing conditions, resulting in differential activation of NMDARs and Ca^2^^+^ influx dynamics that could dictate whether LTP or LTD will be induced. Indeed, GABA has been shown to influence dendritic depolarization and modify the balance of NMDARs and voltage-sensitive Ca^2^^+^ channels at corticostriatal synapses, where it controls the polarity of STDP (Paille et al., 2013). We note, however, that all our experiments were conducted in the absence of GABA_B_ receptor antagonists, thus potential effects of these receptors, especially presynaptic autoreceptors (Davies et al., 1991) in anti-Hebbian t-LTD cannot be excluded.

### PHYSIOLOGICAL RELEVANCE OF ANTI-HEBBIAN t-LTP

The DA hypothesis of reward learning posits that DA serves as an instructing signal that enables and/or facilitates synaptic modifications to reinforce ongoing associative adaptive behaviors and mnemonic processes (Schultz, 2002; Wise, 2004). The profound effects of DA on STDP in the PFC support the emerging tri-component STDP learning rule that neuromodulators can potently influence the gating, polarity, shape, timing window, and other quantitative parameters of STDP (Pawlak et al., 2010). Importantly, our results suggest that the effect of DA is always facilitating, regardless of the temporal order of pre vs. postsynaptic spiking. This provides a mechanism of spatial and temporal binding of active but not necessarily causally correlated inputs to activated DA afferents to strengthen these inputs. Anti-Hebbian t-LTP may serve to strengthen late-spiking inputs which would have been weakened otherwise under Hebbian STDP, attaching necessary motivational salience for these inputs. Prefrontal layer V neurons receive inputs from other cortical regions as well as thalamocortical and hippocampal pathways and process top-down information from these regions. Implementation of both Hebbian and anti-Hebbian t-LTP by these neurons may prove advantageous in the effective association and integration of cortical, thalamus, and hippocampal information to guide behavioral adaptation. However, in computational models that assign importance to STDP for learning and memory, typically generation of both LTP and LTD is considered relevant. Thus, mechanisms that can weaken the potentiated synapses on these neurons should exist. Additional studies will be required to define how timing of DA release, local concentration and dynamics of DA transients, and DA receptor distributions at target dendritic spines shape STDP window and polarity, in particular t-LTD. Incorporating these mechanistic details can improve the current neural network models (Baras and Meir, 2007; Florian, 2007; Izhikevich, 2007; Fremaux et al., 2010) of learning and reward, which in turn, will deepen our understanding of the roles of DA in normal reward and motivation as well as in pathological conditions, such as addiction, depression, and schizophrenia.

## AUTHOR CONTRIBUTIONS

Hongyu Ruan, Taixiang Saur, and Wei-Dong Yao designed research; Hongyu Ruan and Taixiang Saur performed research; Hongyu Ruan, Taixiang Saur, and Wei-Dong Yao analyzed data; and Wei-Dong Yao wrote the paper.

## Conflict of Interest Statement

The authors declare that the research was conducted in the absence of any commercial or financial relationships that could be construed as a potential conflict of interest.

## References

[B1] Auberson Y. P. Allgeier H. Bischoff S. Lingenhoehl K. Moretti R. Schmutz M. (2002). 5-Phosphonomethylquinoxalinediones as competitive NMDA receptor antagonists with a preference for the human 1A/2A, rather than 1A/2B receptor composition. *Bioorg. Med. Chem. Lett.* 12 1099–1102 10.1016/S0960-894X(02)00074-4 11909726

[B2] Baras D. Meir R. (2007). Reinforcement learning, spike-time-dependent plasticity, and the BCM rule. *Neural Comput.* 19 2245–2279 10.1162/neco.2007.19.8.2245 17571943

[B3] Berberich S. Punnakkal P. Jensen V. Pawlak V. Seeburg P. H. Hvalby O. (2005). Lack of NMDA receptor subtype selectivity for hippocampal long-term potentiation. *J. Neurosci.* 25 6907–6910 10.1523/JNEUROSCI.1905-05.2005 16033900 PMC6725356

[B4] Bi G. Q. Poo M. M. (1998). Synaptic modifications in cultured hippocampal neurons: dependence on spike timing, synaptic strength, and postsynaptic cell type. *J. Neurosci.* 18 10464–10472 9852584 10.1523/JNEUROSCI.18-24-10464.1998 PMC6793365

[B5] Bi G. Q. Poo M. M. (2001). Synaptic modification by correlated activity: Hebb’s postulate revisited. *Annu. Rev. Neurosci.* 24 139–166 10.1146/annurev.neuro.24.1.139 11283308

[B6] Bissiere S. Humeau Y. Luthi A. (2003). Dopamine gates LTP induction in lateral amygdala by suppressing feedforward inhibition. *Nat. Neurosci.* 6 587–592 10.1038/nn1058 12740581

[B7] Bliss T. Schoepfer R. (2004). Neuroscience. Controlling the ups and downs of synaptic strength. *Science* 304 973–974 10.1126/science.1098805 15143268

[B8] Caporale N. Dan Y. (2008). Spike timing-dependent plasticity: a Hebbian learning rule. *Annu. Rev. Neurosci.* 31 25–46 10.1146/annurev.neuro.31.060407.125639 18275283

[B9] Cassenaer S. Laurent G. (2012). Conditional modulation of spike-timing-dependent plasticity for olfactory learning. *Nature* 482 47–52 10.1038/nature10776 22278062

[B10] Chen N. Luo T. Raymond L. A. (1999). Subtype-dependence of NMDA receptor channel open probability. *J. Neurosci.* 19 6844–6854 10436042 10.1523/JNEUROSCI.19-16-06844.1999 PMC6782868

[B11] Chiu C. Q. Puente N. Grandes P. Castillo P. E. (2010). Dopaminergic modulation of endocannabinoid-mediated plasticity at GABAergic synapses in the prefrontal cortex. *J. Neurosci.* 30 7236–7248 10.1523/JNEUROSCI.0736-10.2010 20505090 PMC2905527

[B12] Clarke R. J. Johnson J. W. (2006). NMDA receptor NR2 subunit dependence of the slow component of magnesium unblock. *J. Neurosci.* 26 5825–5834 10.1523/JNEUROSCI.0577-06.2006 16723541 PMC6675262

[B13] Couey J. J. Meredith R. M. Spijker S. Poorthuis R. B. Smit A. B. Brussaard A. B. (2007). Distributed network actions by nicotine increase the threshold for spike-timing-dependent plasticity in prefrontal cortex. *Neuron* 54 73–87 10.1016/j.neuron.2007.03.006 17408579

[B14] Cull-Candy S. G. Leszkiewicz D. N. (2004). Role of distinct NMDA receptor subtypes at central synapses. *Sci. STKE* 2004 re16 10.1126/stke.2552004re16 15494561

[B15] Dan Y. Poo M. M. (2006). Spike timing-dependent plasticity: from synapse to perception. *Physiol. Rev.* 86 1033–1048 10.1152/physrev.00030.2005 16816145

[B16] Davies C. H. Starkey S. J. Pozza M. F. Collingridge G. L. (1991). GABA autoreceptors regulate the induction of LTP. *Nature* 349 609–611 10.1038/349609a0 1847993

[B17] Feldman D. E. (2012). The spike-timing dependence of plasticity. *Neuron* 75 556–571 10.1016/j.neuron.2012.08.001 22920249 PMC3431193

[B18] Fino E. Deniau J. M. Venance L. (2008). Cell-specific spike-timing-dependent plasticity in GABAergic and cholinergic interneurons in corticostriatal rat brain slices. *J. Physiol.* 586 265–282 10.1113/jphysiol.2007.144501 17974593 PMC2375545

[B19] Fino E. Glowinski J. Venance L. (2005). Bidirectional activity-dependent plasticity at corticostriatal synapses. *J. Neurosci.* 25 11279–11287 10.1523/JNEUROSCI.4476-05.2005 16339023 PMC6725902

[B20] Florian R. V. (2007). Reinforcement learning through modulation of spike-timing-dependent synaptic plasticity. *Neural Comput.* 19 1468–1502 10.1162/neco.2007.19.6.1468 17444757

[B21] Fremaux N. Sprekeler H. Gerstner W. (2010). Functional requirements for reward-modulated spike-timing-dependent plasticity. *J. Neurosci.* 30 13326–13337 10.1523/JNEUROSCI.6249-09.2010 20926659 PMC6634722

[B22] Fuster J. M. (2008). *The Prefrontal Cortex*. New York: Academic Press

[B23] Ganguly K. Poo M. M. (2013). Activity-dependent neural plasticity from bench to bedside. *Neuron* 80 729–741 10.1016/j.neuron.2013.10.028 24183023

[B24] Gerkin R. C. Lau P. M. Nauen D. W. Wang Y. T. Bi G. Q. (2007). Modular competition driven by NMDA receptor subtypes in spike-timing-dependent plasticity. *J. Neurophysiol.* 97 2851–2862 10.1152/jn.00860.2006 17267756

[B25] Greengard P. Allen P. B. Nairn A. C. (1999). Beyond the dopamine receptor: the DARPP-32/protein phosphatase-1 cascade. *Neuron* 23 435–447 10.1016/S0896-6273(00)80798-9 10433257

[B26] Han V. Z. Grant K. Bell C. C. (2000). Reversible associative depression and nonassociative potentiation at a parallel fiber synapse. *Neuron* 27 611–622 10.1016/S0896-6273(00)00070-2 11055442

[B27] Hebb D. O. (1949). *The Organization of Behavior*. New York: Wiley

[B28] Izhikevich E. M. (2007). Solving the distal reward problem through linkage of STDP and dopamine signaling. *Cereb. Cortex* 17 2443–2452 10.1093/cercor/bhl152 17220510

[B29] Johnston D. Hoffman D. A. Colbert C. M. Magee J. C. (1999). Regulation of back-propagating action potentials in hippocampal neurons. *Curr. Opin. Neurobiol.* 9 288–292 10.1016/S0959-4388(99)80042-7 10395568

[B30] Lamsa K. P. Heeroma J. H. Somogyi P. Rusakov D. A. Kullmann D. M. (2007). Anti-Hebbian long-term potentiation in the hippocampal feedback inhibitory circuit. *Science* 315 1262–1266 10.1126/science.1137450 17332410 PMC3369266

[B31] Lau C. G. Zukin R. S. (2007). NMDA receptor trafficking in synaptic plasticity and neuropsychiatric disorders. *Nat. Rev. Neurosci.* 8 413–426 10.1038/nrn2153 17514195

[B32] Letzkus J. J. Kampa B. M. Stuart G. J. (2006). Learning rules for spike timing-dependent plasticity depend on dendritic synapse location. *J. Neurosci.* 26 10420–10429 10.1523/JNEUROSCI.2650-06.2006 17035526 PMC6674691

[B33] Lin Y. W. Min M. Y. Chiu T. H. Yang H. W. (2003). Enhancement of associative long-term potentiation by activation of beta-adrenergic receptors at CA1 synapses in rat hippocampal slices. *J. Neurosci.* 23 4173–4181 12764105 10.1523/JNEUROSCI.23-10-04173.2003 PMC6741099

[B34] Liu L. Wong T. P. Pozza M. F. Lingenhoehl K. Wang Y. Sheng M. (2004). Role of NMDA receptor subtypes in governing the direction of hippocampal synaptic plasticity. *Science* 304 1021–1024 10.1126/science.1096615 15143284

[B35] Liu Q. S. Pu L. Poo M. M. (2005). Repeated cocaine exposure in vivo facilitates LTP induction in midbrain dopamine neurons. *Nature* 437 1027–1031 10.1038/nature04050 16222299 PMC1457101

[B36] Lu J. T. Li C. Y. Zhao J. P. Poo M. M. Zhang X. H. (2007). Spike-timing-dependent plasticity of neocortical excitatory synapses on inhibitory interneurons depends on target cell type. *J. Neurosci.* 27 9711–9720 10.1523/JNEUROSCI.2513-07.2007 17804631 PMC6672961

[B37] Magee J. C. Johnston D. (1997). A synaptically controlled, associative signal for Hebbian plasticity in hippocampal neurons. *Science* 275 209–213 10.1126/science.275.5297.209 8985013

[B38] Malenka R. C. Bear M. F. (2004). LTP and LTD: an embarrassment of riches. *Neuron* 44 5–21 10.1016/j.neuron.2004.09.012 15450156

[B39] Markram H. Lubke J. Frotscher M. Sakmann B. (1997). Regulation of synaptic efficacy by coincidence of postsynaptic APs and EPSPs. *Science* 275 213–215 10.1126/science.275.5297.213 8985014

[B40] Massey P. V. Johnson B. E. Moult P. R. Auberson Y. P. Brown M. W. Molnar E. (2004). Differential roles of NR2A and NR2B-containing NMDA receptors in cortical long-term potentiation and long-term depression. *J. Neurosci.* 24 7821–7828 10.1523/JNEUROSCI.1697-04.2004 15356193 PMC6729941

[B41] Meredith R. M. Floyer-Lea A. M. Paulsen O. (2003). Maturation of long-term potentiation induction rules in rodent hippocampus: role of GABAergic inhibition. *J. Neurosci.* 23 11142–11146 14657173 10.1523/JNEUROSCI.23-35-11142.2003 PMC6741060

[B42] Morishita W. Connor J. H. Xia H. Quinlan E. M. Shenolikar S. Malenka R. C. (2001). Regulation of synaptic strength by protein phosphatase 1. *Neuron* 32 1133–1148 10.1016/S0896-6273(01)00554-2 11754843

[B43] Morishita W. Lu W. Smith G. B. Nicoll R. A. Bear M. F Malenka R. C. (2007). Activation of NR2B-containing NMDA receptors is not required for NMDA receptor-dependent long-term depression. *Neuropharmacology* 52 71–76 10.1016/j.neuropharm.2006.07.005 16899258

[B44] Mrzljak L. Bergson C. Pappy M. Huff R. Levenson R Goldman-Rakic P. S. (1996). Localization of dopamine D4 receptors in GABAergic neurons of the primate brain. *Nature* 381 245–248 10.1038/381245a0 8622768

[B45] Mulkey R. M. Endo S. Shenolikar S. Malenka R. C. (1994). Involvement of a calcineurin/inhibitor-1 phosphatase cascade in hippocampal long-term depression. *Nature* 369 486–488 10.1038/369486a0 7515479

[B46] Paille V. Fino E. Du K. Morera-Herreras T. Perez S. Kotaleski J. H. (2013). GABAergic circuits control spike-timing-dependent plasticity. *J. Neurosci.* 33 9353–9363 10.1523/JNEUROSCI.5796-12.2013 23719804 PMC6618570

[B47] Pawlak V. Kerr J. N. (2008). Dopamine receptor activation is required for corticostriatal spike-timing-dependent plasticity. *J. Neurosci.* 28 2435–2446 10.1523/JNEUROSCI.4402-07.2008 18322089 PMC6671189

[B48] Pawlak V. Wickens J. R. Kirkwood A. Kerr J. N. (2010). Timing is not everything: neuromodulation opens the STDP gate. *Front. Synaptic Neurosci.* 2:146 10.3389/fnsyn.2010.00146 PMC3059689 21423532

[B49] Riccio A. Ginty D. D. (2002). What a privilege to reside at the synapse: NMDA receptor signaling to CREB. *Nat. Neurosci.* 5 389–390 10.1038/nn0502-389 11976696

[B50] Safo P. K. Regehr W. G. (2005). Endocannabinoids control the induction of cerebellar LTD. *Neuron* 48 647–659 10.1016/j.neuron.2005.09.020 16301180

[B51] Schultz W. (2002). Getting formal with dopamine and reward. *Neuron* 36 241–263 10.1016/S0896-6273(02)00967-4 12383780

[B52] Seamans J. K. Durstewitz D. Christie B. R. Stevens C. F. Sejnowski T. J. (2001). Dopamine D1/D5 receptor modulation of excitatory synaptic inputs to layer V prefrontal cortex neurons. *Proc. Natl. Acad. Sci. U.S.A.* 98 301–306 10.1073/pnas.98.1.301 11134516 PMC14585

[B53] Seamans J. K. Yang C. R. (2004). The principal features and mechanisms of dopamine modulation in the prefrontal cortex. *Prog. Neurobiol.* 74 1–58 10.1016/j.pneurobio.2004.05.006 15381316

[B54] Seol G. H. Ziburkus J. Huang S. Song L. Kim I. T. Takamiya K. (2007). Neuromodulators control the polarity of spike-timing-dependent synaptic plasticity. *Neuron* 55 919–929 10.1016/j.neuron.2007.08.013 17880895 PMC2756178

[B55] Shen W. Flajolet M. Greengard P. Surmeier D. J. (2008). Dichotomous dopaminergic control of striatal synaptic plasticity. *Science* 321 848–851 10.1126/science.1160575 18687967 PMC2833421

[B56] Tully K. Li Y. Tsvetkov E. Bolshakov V. Y. (2007). Norepinephrine enables the induction of associative long-term potentiation at thalamo-amygdala synapses. *Proc. Natl. Acad. Sci. U.S.A.* 104 14146–14150 10.1073/pnas.0704621104 17709755 PMC1955781

[B57] Varela J. A. Hirsch S. J. Chapman D. Leverich L. S. Greene R. W. (2009). D1/D5 modulation of synaptic NMDA receptor currents. *J. Neurosci.* 29 3109–3119 10.1523/JNEUROSCI.4746-08.2009 19279248 PMC2684496

[B58] Wang H. Stradtman G. G. III Wang X. J. Gao W. J. (2008). A specialized NMDA receptor function in layer 5 recurrent microcircuitry of the adult rat prefrontal cortex. *Proc. Natl. Acad. Sci. U.S.A.* 105 16791–16796 10.1073/pnas.0804318105 18922773 PMC2575498

[B59] Weitlauf C. Honse Y. Auberson Y. P. Mishina M. Lovinger D. M Winder D. G. (2005). Activation of NR2A-containing NMDA receptors is not obligatory for NMDA receptor-dependent long-term potentiation. *J. Neurosci.* 25 8386–8390 10.1523/JNEUROSCI.2388-05.2005 16162920 PMC6725680

[B60] Wigstrom H. Gustafsson B. (1983). Facilitated induction of hippocampal long-lasting potentiation during blockade of inhibition. *Nature* 301 603–604 10.1038/301603a0 6298626

[B61] Wise R. A. (2004). Dopamine, learning and motivation. *Nat. Rev. Neurosci.* 5 483–494 10.1038/nrn1406 15152198

[B62] Xu T. X. Yao W. D. (2010). D1 and D2 dopamine receptors in separate circuits cooperate to drive associative long-term potentiation in the prefrontal cortex. *Proc. Natl. Acad. Sci. U.S.A.* 107 16366–16371 10.1073/pnas.1004108107 20805489 PMC2941310

[B63] Xu T. X. Sotnikova T. D. Liang C. Zhang J. Jung J. U. Spealman R. D. (2009). Hyperdopaminergic tone erodes prefrontal long-term potential via a D2 receptor-operated protein phosphatase gate. *J. Neurosci.* 29 14086–14099 10.1523/JNEUROSCI.0974-09.2009 19906957 PMC2818669

[B64] Zhang J. C. Lau P. M. Bi G. Q. (2009). Gain in sensitivity and loss in temporal contrast of STDP by dopaminergic modulation at hippocampal synapses. *Proc. Natl. Acad. Sci. U.S.A.* 106 13028–13033 10.1073/pnas.0900546106 19620735 PMC2713390

[B65] Zhao M. G. Toyoda H. Lee Y. S. Wu L. J. Ko S. W. Zhang X. H. (2005). Roles of NMDA NR2B subtype receptor in prefrontal long-term potentiation and contextual fear memory. *Neuron* 47 859–872 10.1016/j.neuron.2005.08.014 16157280

